# Serial cross-sectional school surveys identifies C469Y, P553L, R561H and A675V kelch 13 mutations associated with artemisinin resistance in Western Kenya

**DOI:** 10.1038/s41598-025-22286-7

**Published:** 2025-11-03

**Authors:** Victor Osoti, Kevin Wamae, Moses M. Musau, John B. Magudha, Leonard Ndwiga, Paul M. Gichuki, Collins Okoyo, Kiplagat Rosebella, Sammy Mahugu, Stephen Aricha, Regina Kandie, Kibor Keitany, Stella Kepha, Charles Mwandawiro, Philip Bejon, Robert W. Snow, L. Isabella Ochola-Oyier

**Affiliations:** 1 Centre for Geographic Medicine Research (Coast), Kenya Medical Research Institute-Wellcome Trust Research Programme, Kilifi, Kenya; 2 Eastern and Southern Africa Centre of International Parasite Control, Kenya Medical Research Institute, Nairobi, Kenya; 3 Department of Epidemiology, Statistics and Informatics, Kenya Medical Research Institute, Nairobi, Kenya; 4 Centre for Tropical Medicine and Global Health, Nuffield Department of Medicine, University of Oxford, Oxford, UK; 5 Division of National Malaria Programme, Ministry of Health, Nairobi, Kenya

**Keywords:** Malaria, Drug resistance, Artemisinin, R561H, A675V, C469Y, P553L, Amplicon, Deep-sequencing, Kenya, Genetics, Microbiology, Molecular biology

## Abstract

Recent reports from East African countries indicate the emergence and spread of artemisinin partial resistance (ART-R), posing a significant threat to malaria control efforts in the region. The presence of critical *Plasmodium falciparum* kelch13 (k13) resistance markers, including *C469Y*, *P553L* and *A675V*, have been detected in Kenya, although their clinical significance remains unclear. This highlights an urgent need to closely monitor the prevalence of these mutations. A total of 24,227 dried blood spot (DBS) samples were collected from 82 primary schools across eight counties in Western Kenya during repeated cross-sectional surveys conducted in 2019 (*n* = 8,111), 2022 (*n* = 8,086), and 2023 (*n* = 8,200). Initial screening was performed using a rapid diagnostic test (RDT), and DNA extraction was conducted on RDT-positive samples. These samples were further analyzed using a *Pf*18s qPCR assay to quantify the *Plasmodium falciparum* DNA. Amplicons from malaria-positive samples were sequenced using a previously established amplicon deep sequencing pipeline to analyze mutations in *Pfk13*. A total of 500, 920 and 1058 samples from 2019, 2022, and 2023, respectively were successfully processed, enabling a temporal assessment of the changes in k13 mutations in the region. Four mutations that have previously been associated with artemisinin resistance were found. The A675V mutation was the most prevalent, being found in all 8 counties. It was absent in 2019 and increased from 0.9% in 2022 to 5% in 2023. In contrast, the C469Y mutation declined from 4% in 2022 to 1% in 2023, maintaining a presence in 3 counties. The P553L mutation was only detected in 2022 in 1.2% of the samples across 5 counties. The R561H mutation was not detected in 2019 and 2022 but emerged at a low frequency (0.5%) in 2023 in 2 counties. Siaya and Kisumu were the only counties with all 4 validated mutations between 2022 and 2023. The rising prevalence and geographical presence of the A675V mutation and the new detection of R561H in 2023 highlights the critical need for robust molecular surveillance systems to track the frequency and geographic spread of resistance markers. School-based sampling presents a practical and scalable approach for molecular surveillance, providing early warning signals for potential resistance hotspots. Additionally, the detection of the four WHO validated*Pfk13* artemisinin resistance mutations in Western Kenya underscores the urgency of conducting regular Therapeutic Efficacy Studies (TES) to assess the continued efficacy of frontline antimalarial treatments. Integrating molecular surveillance with TES will generate important data to inform national treatment policies and support the long-term effectiveness of malaria control strategies in Kenya.

**Supplementary Information:**

The online version contains supplementary material available at 10.1038/s41598-025-22286-7.

## Introduction

As malaria^1^ cases decline across the African continent^2^, the emergence of artemisinin resistance is one of several biological threats for future reductions in the malaria burden. The emergence of confirmed clinical artemisinin resistance (ART-R) in Africa, currently documented in four countries (Eritrea, Rwanda, Uganda and United Republic of Tanzania) and is suspected in Ethiopia, the Sudan, Namibia and Zambia presents a significant challenge for malaria control efforts on the continent, which bears over 95% of the global malaria burden^2^. Countries in East Africa and the Horn of Africa, including Eritrea, Ethiopia, Rwanda, Tanzania and Uganda, are grappling with a high prevalence of kelch13 (*Pfk13*) mutations, a molecular marker of artemisinin resistance, across multiple sites^3–6^. Neighboring nations, such as Kenya^7–9^, Democratic Republic of Congo^10,11^ and Zambia^12^, have also recently identified these mutations, albeit at lower prevalence, highlighting a growing regional concern.

Artemisinin resistance in *Plasmodium falciparum* malaria was first suspected in western Cambodia in the early 2000 s, with clinical impact becoming evident by 2004^13,14^. This resistance was largely driven by the emergence of the C580Y mutation in the *Pfk13* gene^15^. Since then, it has either spread or independently emerged in various parts of Cambodia, Thailand, Vietnam, Myanmar, and Laos^16^.

In contrast, studies from East Africa have identified distinct *k13* mutations as drivers of artemisinin partial resistance, independent of the Asian mutations. In Rwanda, the R561H mutation has been implicated^17^, while Uganda has reported C469Y and A675V mutations^18^. In Tanzania, nationwide malaria molecular surveillance revealed a high prevalence of R561H mutation, a validated artemisinin resistance *k13* mutation, in the Kagera region of northwestern Tanzania. Supporting these findings, a Therapeutic Efficacy Study (TES) conducted in Karagwe District found the mutation in over 20% of patients, with a strong association with delayed parasite clearance. Additionally, day 3 parasitemia exceeded the World Health Organization (WHO) 5% threshold for suspected artemisinin resistance (ART-R), underscoring growing concerns about emerging resistance in the region^19^. Haplotype analysis suggested that some of these parasites are related to isolates that were collected in Rwanda in 2015, supporting the regional spread of the R561H mutation. Additionally, other validated k13 resistance polymorphisms, including A675V and R622I, have also been identified^20^. R561H, C469Y, and P441L mutations have been detected at low frequencies in the Democratic Republic of Congo (DRC), specifically in regions bordering Uganda and Rwanda^4,11^. previously reported a significant increase in the prevalence of *PfK13* mutations in Uganda. In Northern Uganda, the prevalence of the C469Y and A675V mutations reached up to 50%, while sites in Southern Uganda documented a 40% prevalence of the C469F mutation in 2018. *Pfk13* mutations (469 F, 561 H, and 675 V) associated with artemisinin resistance, are increasingly prevalent in southern Rwanda. The prevalence of these mutations rose from 9.1% in 2019 to 17.5% by 2023, indicating a concerning upward trend in resistance markers in Rwanda^21^.

Cross-sectional surveys are valuable tools for the surveillance of antimalarial drug resistance, enabling the monitoring of various *Plasmodium falciparum* resistance markers to different antimalarial therapies^11^. Cross-sectionals surveys should be repeated in the same locations to examine temporal changes in emerging resistance markers.

Single nucleotide polymorphisms (SNPs) in the *Pfk13* gene are linked to varying levels of susceptibility to components of artemisinin-based combination therapies (ACTs)^22,23^. The current, pressing concern of artemisinin-resistant mutations across the Horn and East Africa, necessitates a scaled molecular surveillance in the region. School surveys are a convenient sample set to determine the circulating parasite genotypes in the population. We conducted a surveillance of *kelch13* mutations over time on samples collected from Western Kenya in 2019, 2022, and 2023 to assess the frequency and temporal changes of *Pfk13* mutations associated with antimalarial drug resistance.

## Materials and methods

### Study area and sampling

**Fig. 1:**
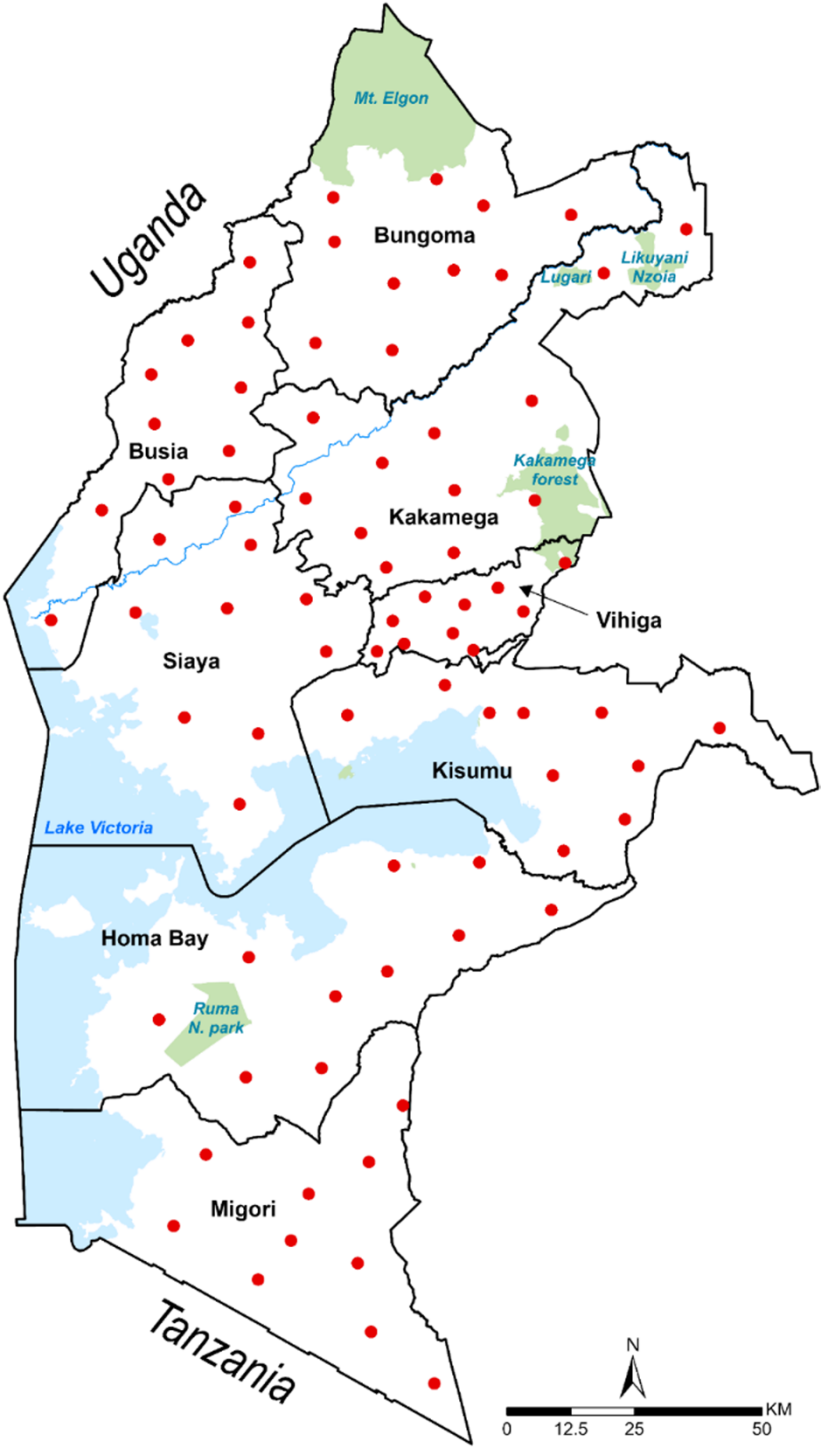
The map of Western Kenya showing schools sampled in 2019, 2022, 2023. The red dots indicate the schools from which malaria-positive children were identified by a malaria rapid diagnostic test, and a dried blood spot was collected from each child.

Kenya exhibits a range of malaria transmission patterns, with the highest levels of perennial transmission occurring in the densely populated eight counties around Lake Victoria in Western Kenya^24^. Since 2010, these counties have been prioritized for targeted malaria control interventions. Since 2019, the Kenya Medical Research Institute Eastern and Southern Africa Centre of International Parasite Control (KEMRI ESACIPAC) in collaboration with KEMRI-Wellcome Trust Research Programme (KWTRP) has led efforts to assess national school-based malaria parasite prevalence surveys to augment periodic national malaria indicator surveys and support modelling of transmission intensity over time. A total of 82 primary schools were selected for sampling across eight counties in Western Kenya, namely Migori, Homa Bay, Kisumu, Siaya, Busia, Kakamega, Vihiga, and Bungoma **(**Fig. 1**).** Cross-sectional surveys were conducted in the same schools across three time-points (2019, 2022, and 2023) to allow for meaningful comparisons over time. At each of these schools, the study team randomly selected 20 students per class aged between 5 and 15 years, comprising 10 boys and 10 girls from classes ranging from Pre-Primary 2 (PP2) to Grade 6, representing 100 children per school for each survey year. The schools included in the study were relatively similar in size. Each child provided a finger-prick blood sample for a rapid diagnostic test (RDT) using CareStart™ Malaria kits, along with a ~ 50µL blood sample collected on Whatman™ CF12 filter papers, (Cytiva, USA). The filter papers were air-dried for at least one hour, sealed in zip-lock bags and shipped to the KWTRP laboratories for further analysis. We leveraged an existing study protocol originally developed for a national school-based deworming monitoring and evaluation exercise, which included an assessment of malaria infection among primary school children in eight counties in Western Kenya. An amendment to the protocol was made to incorporate the collection of dried blood spot (DBS) samples for artemisinin resistance genotyping. The age group targeted in the original protocol was based on the objectives of the deworming and malaria prevalence components and was not specifically selected for the artemisinin resistance genotyping.

The study received ethical approval from the KEMRI Scientific and Ethics Review Unit (SERU) (Approval Number: KEMRI/SERU/ESACIPAC/11/3822). Consent was obtained from selected county ministries of health and education, heads of schools briefed students and parents who were given the opportunity to opt out and children could refuse participation on the day of the survey. Prior to sample collection, study staff administered a questionnaire to determine if participants had a fever. Students who were febrile or unwell at the time were excluded from the study.

Children who tested positive for malaria were treated with artemether-lumefantrine, following the national treatment guidelines, and written instructions on dosing were provided to both the child and their class teacher^25^.

### DNA extraction, sequencing and data analysis

Parasite DNA was extracted from RDT-positive DBS samples using a previously published protocol^25^. Four punches, 6 mm each, were punched from two locations (at the center and periphery) of the DBS into a 1.5 ml Eppendorf tube. The DBS punching was performed with a BSD600 Ascent A2 Semi-Automated Puncher (BSD Robotics, Australia) that was cleaned using a cotton swab soaked in 100% ethanol between each run and cross-contamination between samples was mitigated by punching a blank filter card four times after each sample. DNA extraction was done using the Chelex saponin method^25^. Parasite DNA was amplified using 18 s rRNA *Plasmodium falciparum* qPCR assay^25^. Samples with a cycling threshold (Ct) value of less than 36 were selected for PCR amplification and sequencing. The *Pfk13* amplicons (Supplementary Table 2) were generated in a 5-amplicon sequencing panel assay utilized in the KWTRP lab and thus also included *Pfdhfr*, *Pfdhps*, *Pfmdr1* and *Pfama1* amplicons using primers labeled with molecular identifiers (MIDs), as described by^25^. PCR amplicons with non-overlapping molecular identifiers (MIDs) were combined to form distinct amplicon pools. Each pool underwent purification using the Zymo ZR-96 DNA Clean & Concentrator-5 Kit (ZR D4014, Zymo Research) following the manufacturer’s instructions. The purified amplicons were then eluted in 30 µl of PCR-grade water. To assess the DNA concentration, we used the Qubit™ 1X dsDNA High Sensitivity (HS) Assay Kit (Invitrogen) according to the manufacturer’s instructions. Subsequently, the purified PCR amplicons were normalized to a final concentration of 200 ng/µl each using PCR-grade water.

Library preparation was carried out using the KAPA kit, followed by size selection using 1X AMPure XP beads. Adapter-ligated libraries were amplified with Illumina primers and underwent a second clean-up using 0.8X AMPure beads. The resulting libraries were quantified with the Qubit™ 1X dsDNA High Sensitivity (HS) Assay Kit (Invitrogen) and size-verified using the Agilent 2200 TapeStation system (Agilent Technologies). Libraries were then pooled in equimolar ratios, denatured, and spiked with 15% PhiX DNA for quality control. The amplicon libraries were sequenced at the International Livestock Research Institute using the MiSeq reagent kit v3 (Illumina) on the MiSeq. The entire protocol has been outlined by^25^. Data analysis was performed using SeekDeep v3.0.1, as described in^9^. Due to the expected high frequency of the well-described *Pfdhfr*, *fdhps* and *Pfmdr1* mutations, these genes also served as a control in the bioinformatic pipeline analysis. However, this manuscript only presents the *Pfk13* data. All statistical analyses were conducted in R (version 4.4.3; R Core Team, 2024), comparisons were conducted with samples from Western Kenya collected in 2019^25^ and 2022^25^.

## Results

### Detection of WHO validated mutations

Between March and April in 2019, 2022, and 2023, repeated cross-sectional surveys were conducted to assess malaria prevalence among primary school children across eight malaria-endemic counties in Western Kenya: Migori, Homa Bay, Kisumu, Siaya, Busia, Bungoma, Vihiga, and Kakamega. A total of 24,227 children were sampled and screened for *Plasmodium falciparum* infection using RDTs. DNA was extracted from all RDT-positive samples, followed by 18 s *P. falciparum*

qPCR. Samples with a Ct value of less than 36 were subsequently processed for PCR amplicon generation and sequencing.

In 2019, 8,111 children aged 4–18 years were screened, of whom 2,247 (27.7%) tested positive by RDT. Subsequent 18 s *Pf* qPCR analysis detected *P. falciparum* DNA in 1,263 samples (56.2% of RDT-positive cases) and 500 samples were selected for genotyping. In 2022, 8,086 children aged 5–14 years were sampled, with 1,573 (19.5%) testing positive by RDT. *P. falciparum* DNA was detected in 1,260 of the RDT positive samples (80.1%) using 18 S *Pf* qPCR, and 920 samples were selected for genotyping to investigate drug resistance markers. In 2023, 8,200 children between 5 and 14 years of age were screened, 2,226 (27.1%) children were RDT positive for malaria while 1370 (61.5%) of the RDT positive samples had detectable DNA material using 18 s *Pf* qPCR assay, and 1,058 qPCR-confirmed samples were selected for genotyping to assess *P. falciparum* drug resistance markers.

For samples collected during the 2019, 2022, and 2023 surveys, sequencing of the *k13* gene (covering amplicon fragments 2156–2645 bp) (Supplementary Fig. 1) was successfully completed for 196 samples (39.2%) in 2019, 523 samples (57%) in 2022, and 727 samples (69%) in 2023. For the second fragment (2509–3020 bp) (Supplementary Fig. 1), sequencing was successfully performed on 110 samples (12%) in 2022 and 965 samples (91%) in 2023. The second fragment was not amplified in samples collected in 2019 (Table 1). Several mutations were detected in the *pfk13* gene, including validated markers of artemisinin partial resistance (Table 1). Four validated *k13* mutations associated with artemisinin resistance were identified: C469Y; P553L; R561H; and A675V. All these mutations were identified in mixed genotype infections.

### Prevalence of the WHO validated mutations

The C469Y mutation was observed in a total of 8 samples (1.1%) and present in 4 counties in 2023, with Bungoma having the largest number (3) of mutant samples (Fig. 1). The overall proportion of this mutation decreased from 4% in 2022 to 1% in 2023. However, a county level analysis revealed an increase in Bungoma to 7.7% and a reduction in Migori, 1%, and Siaya, 3%, from 5.9%, 2.1% and 6.9% in 2022, respectively. It was only observed in Kakamega at 1.3% in 2023. P553L was only observed in 2022 in 5 counties, with no mutations in 2023 **(**Fig. 1**).** The R561H/C mutation was not present in 2019 and 2022 but appeared in 2023 at a frequency of 0.9% **(**Table 1**).** Both R561H and R561C mutations were only identified in Kisumu and Siaya counties **(**Fig. 1**)**, while Bungoma and Busia were the only counties with the R561C mutation. The R561H mutation was found in 5 samples (0.5%) in 2023.

**Fig. 2:**
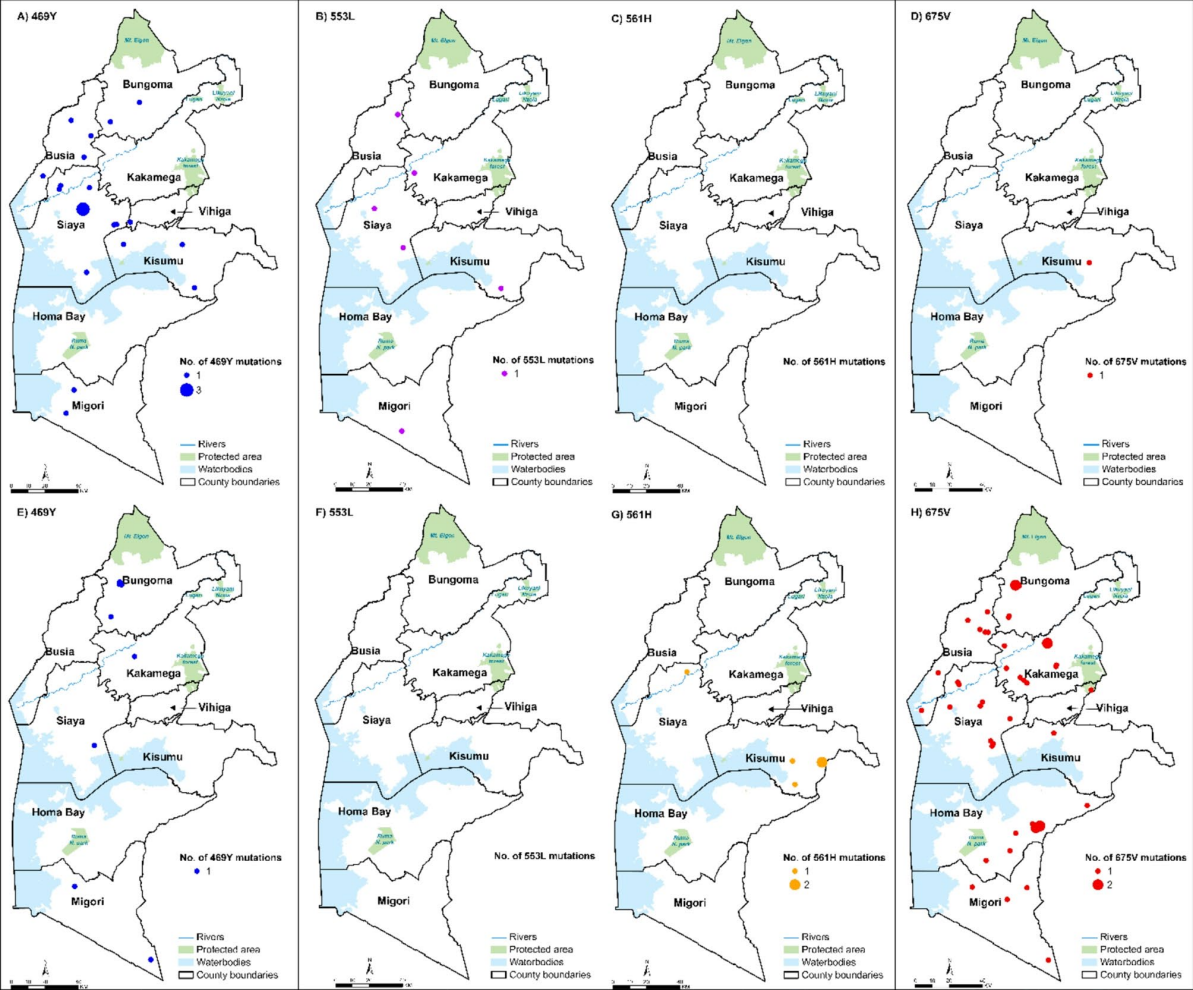
Spatial distribution of k13 validated resistance mutations by village in Western Kenya. The 8 counties have been demarcated and labelled, and each dot represents the village of the child harboring the parasite with the mutation. The size of the dot varies depending on the number of mutations identified in that village. **A-D** illustrates mutations that were identified in 2022 while **E-H** shows mutations that were identified in 2023. Each color shows a different mutation. No mutations were identified in 2019.

All 8 counties harbored the A675V **(**Fig. 2**)** mutation in 2023, as the dominant mutation in 48 samples (5%), with Homa Bay (which in prior years had no mutations) topping the list with 11 mutant samples followed by Siaya (10) and thereafter Busia (7).

### Frequency of other k13 mutations

The prevalence of most *Pfk13* SNPs was low **(**Table 1**)**, while the frequencies of the wild type remained high (> 96%) across the years, suggesting limited accumulation of k13 SNPs. An additional twenty non-synonymous mutations **(**Table 1**)** were detected. A578S was a consistent SNP that fluctuated over time as mixed infections, increasing from 7.7% (2019) to 15.3% (2022) and declining to 6% (2023). Mutations such as S522C and P667A/S (not significantly associated k13 mutations) were identified in both 2022 and 2023, and P441A (a candidate k13 mutation) was only observed in 2022. E691D was only observed in 2022 at a high prevalence in mixed infections (89.1%). There were a higher number of k13 mutation loci (codons) in 2022 of 18 compared to 2 in 2019 and 13 in 2023, (Table 1) across 315, 17 and 208 samples, respectively **(**Table 2**).** Bungoma, Busia, Siaya and Kisumu counties contained *Pfk13* SNPs in all 3 years.

In 2023, the highest proportion of *Pfk13* SNPs was observed in Migori (21%), which also contained the third largest number of *Pfk13* SNPs over the entire sampling period, Siaya (16%), Homa Bay (15%) and Kakamega (14%). Notably, all 3 counties except for Siaya showed an increase in SNP frequency over the 3-year sampling period (Supplementary Table 1). Similar to Siaya, Busia and Bungoma counties had less *Pfk13* SNPs by 2023, however overall, Siaya and Busia had the highest number of *Pfk13* SNPs in the population. Vihiga was unique as it consistently recorded low SNP frequencies (≤ 4%) across all years (Table 2) with a reduction by half in the number of unique *Pfk13* mutations between 2022 and 2023 (Table 3). The proportion of unique *Pfk13* SNPs per county was maintained in Kisumu, with all other counties showing a reduction in 2023 (Table 3).

**Table 1:** The frequency of k13mutations from the schools’ surveys conducted in 2019, 2022 and 2023 in Western Kenya.

	Codon Position	Reference Allele	Non-Reference Allele	Non-reference allele frequencies		
				2019	2022	2023
**k13**	441	P	A	0	6/523 (1.1%)	0
	453	G	C	0	7/523 (1.3%)	0
	**469**	C	Y	0	21/523 (4%)	8/727 (1.1%)
	471	R	C	0	0	8/727 (1.1%)
	489	N	K	0	12/523 (2.3%)	24/727 (3.3%)
	499	N	S	0	5/523 (1%)	0
	501	D	Y	0	0	6/727 (0.8%)
	504	A	V	0	6/523 (1.1%)	0
	517	V	I	0	9/523 (1.7%)	0
	522	S	C	0	18/523 (3.4%)	13/727 (1.8%)
	537	N	S	0	2/523 (0.4%)	0
	**553**	P	S	0	6/523 (1.2%)	0
		P	L	0	6/523 (1.2%)	0
	558	Y	C	0	14/523 (2.7%)	0
	**561**	R	H	0	0	5/965 (0.5%)
		R	C	0	0	4/965 (0.4%)
	569	A	T	0	11/523 (2.1%)	7/965 (0.7%)
		A	S	2/196 (1%)	0	5/965 (0.5%)
	578	A	S	15/196 (7.7%)	80/523 (15.3%)	61/965 (6%)
	667	P	A	0	8/110 (7.3%)	1/965 (0.1%)
		P	S	0	0	38/965 (3.9%)
	**675**	A	V	0	1/110 (0.9%)	48/965 (5%)
	679	S	T	0	3/110 (2.7%)	0
	691	E	D	0	98/110 (89.1%)	0

*110 (2022) and 965 (2023) samples were successfully sequenced using primers targeting the second k13 fragment. Codons in bold are the WHO validated artemisinin resistance mutations.

**Table 2:** Total number of individuals with k13 mutations identified in 2019, 2022 and 2023.

Total number of k13 mutations				
	2019	2022	2023	Total
Vihiga	0	19/315 (6.0)	7/208 (3.4)	26
Kakamega	0	15/315 (4.7)	29/208 (13.9)	43
Homa Bay	0	18/315(5.7)	32/208 (15.4)	44
Migori	0	41/315 (13.0)	44/208 (21.2)	50
Kisumu	1/17 (5.9)	26/315 (8.3)	24/208 (11.5)	51
Bungoma	3/17(17.6)	22/315 (7.0)	18/208 (8.7)	85
Busia	5/17 (29.4)	76/315 (24.1)	21/208 (10.1)	102
Siaya	8/17 (47.1)	98/315 (31.1)	33/208 (15.9)	139

**Table 3:** Total number of unique K13 mutations identified in 2019, 2022 and 2023.

Total number of unique k13 mutations			
	2019	2022	2023
**Vihiga**	0	9/24 (37.5)	4/24 (16.7)
**Kakamega**	0	9/24 (37.5)	7/24 (29.2)
**Homa Bay**	0	8/24 (33.3)	7/24 (29.2)
**Migori**	0	13/24 (54.2)	8/24 (33.3)
**Kisumu**	1/24 (4.2)	10/24 (41.7)	10/24 (41.7)
**Bungoma**	1/24 (4.2)	8/24 (33.3)	7/24 (29.2)
**Busia**	1/24 (4.2)	14/24 (58.3)	7/24 (29.2)
**Siaya**	2/24 (8.3)	15/24 (62.5)	9/24 (37.5)

The percentages were calculated over the total number (24) of segregating sites identified between 2019 and 2023.

## Discussion

In 2023 in Western Kenya, three WHO validated *Pfk13* mutations were identified, including an emerging R561H/C mutation not previously observed in this area. Over the one-year study period, we observed an increase and broader geographical distribution of the A675V mutation within the asymptomatic malaria population. The presence of the A675V mutation at 5% in 2023 is worrying and warrants attention, since it has been observed in school-aged children who are an important reservoir for malaria transmission. Furthermore, there is a need to expand the molecular surveillance, to incorporate RDT or microscopy positive cases at health facilities, to better assess prevalence and potential public health implications. The C469Y mutation continues to be maintained though it was at a higher prevalence than A675V in 2022, and reduced in frequency in 2023, comparable to observations made in Uganda^18,26^. The frequency of A675V rapidly rose to become the major *Pfk13* variant in Uganda. In addition to the low C469Y prevalence, the emergent R561H mutation in the Kenyan population requires continuous monitoring to assess whether these low frequency mutations continue to be maintained in prospective studies. R561H has previously been documented in neighboring countries at high prevalence in symptomatic infections in Uganda^4^, Rwanda^17^ and Tanzania’s region bordering Rwanda^27^, highlighting its regional significance. What is intriguing is the reduction in prevalence of C469Y, although our limited sample sizes may have led to errors in measuring rare SNPs. It is important to note that the sequencing success rate in 2019 was substantially lower than in subsequent years, with only 196 samples successfully sequenced for the first fragment and no amplification of the second fragment. This lower yield, combined with the smaller sample size, may have reduced our ability to detect low-frequency variants in 2019 compared to 2022 and 2023.

Annual surveillance remains an important approach to track the emergence and spread of mutations, notably counties bordering western Uganda, northwestern Tanzania and those with high volume human population movement with neighboring countries with high mutation rates. Our findings highlight evolving patterns in both the prevalence and diversity of *Pf*k13 mutations in Western Kenya, potentially signaling emerging or declining selection pressures in different regions. Further whole genome analyses are required to determine the origins of these *Pfk13* WHO validated mutations.

The data provided here corroborates the history of challenges in antimalarial drug use in malaria-endemic regions, clearly demonstrating that following the introduction of a new antimalarial, the emergence and spread of *P. falciparum* resistance mutations is inevitable. This was not previously possible with chloroquine resistance (CQR) since the causal variant in the genetic marker *P. falciparum* chloroquine resistance transporter was described in 2001^28^ years after CQR was widespread globally^29^. Similarly, for SP resistance the early use (in the 1930 s) of sulpha based drugs exacerbated the development of SP resistance development^30^. The advent of whole genome sequencing^31^ hastened the process of identifying genetic markers of resistance, i.e. *Pfk13*^22^. All these mutations are now better tracked and are rapidly informing malaria treatment strategies. Based on this current data, ongoing and more rapid surveillance of resistance markers becomes essential and requires support from public health policy makers to ensure it is sustainable and it immediately feeds back into public health interventions. As a matter of urgency, the efficacy of the ACTs currently in use in Kenya needs to be examined.

## Supplementary Information

Below is the link to the electronic supplementary material.

Supplementary Material 1

Supplementary Material 2

## Data Availability

For data access inquiries, please contact the KEMRI-Wellcome Trust Research Programme Data Governance Committee at dgc@kemri-wellcome.org. Specific requests related to school-based mRDT data should be directed to RWS, while SNP data inquiries can be addressed to LIO. The datasets from the surveillance of *kelch13* mutations linked to artemisinin resistance in Western Kenya are publicly available. The 2023 nucleotide sequence dataset is publicly available in GenBank under accession numbers *Pf k13*_675 (PV776796–PV776815) and *Pf k13***_**469 (PV776816–PV776831). FASTQ files for the K13_469 and K13_675 fragments can be accessed via the Harvard Dataverse (Kenyansa, Victor. 2025. 10.7910/DVN/QVHDSR). The 2022 survey data, also available on Harvard Dataverse, include FASTQ files for the k13-675 fragment (10.7910/DVN/Q7PD5P), FASTA files (10.7910/DVN/O82JXI) and FASTQ files for k13-469 fragment 10.7910/DVN/2TJH9F, with nucleotide sequences for k13-469 submitted to GenBank under accession numbers PQ283632–PQ283660. Additionally, amplicon sequence data from the 2019 survey are available in GenBank under accession numbers OM370918–OM370923 for *Pfk13*.All data are shared under the Creative Commons Attribution 4.0 International License (CC-BY 4.0).
