# Supplementary material for: Serial cross-sectional school surveys identifies C469Y, P553L, R561H and A675V kelch 13 mutations associated with artemisinin resistance in Western Kenya
Source: Sci Rep. 2025 Nov 3;15:38303. doi: 10.1038/s41598-025-22286-7 (PMC12583745; doi:10.1038/s41598-025-22286-7)
Supplement: Supplementary file 1 — Supplementary Material 1 [file 41598_2025_22286_MOESM1_ESM.docx]

**Supplementary Table1: Distribution of k13 mutations detected in the population (2019–2023)**

|  | **Bungoma** | | | **Busia** | | | **Homa Bay** | | | **Kakamega** | | | **Kisumu** | | | **Migori** | | | **Siaya** | | | **Vihiga** | | |
| --- | --- | --- | --- | --- | --- | --- | --- | --- | --- | --- | --- | --- | --- | --- | --- | --- | --- | --- | --- | --- | --- | --- | --- | --- |
|  | **2019** | **2022** | **2023** | **2019** | **2022** | **2023** | **2019** | **2022** | **2023** | **2019** | **2022** | **2023** | **2019** | **2022** | **2023** | **2019** | **2022** | **2023** | **2019** | **2022** | **2023** | **2019** | **2022** | **2023** |
| P441A | 0 | 0 | 0 | 0 | 1 | 0 | 0 | 0 | 0 | 0 | 0 | 0 | 0 | 1 | 0 | 0 | 2 | 0 | 0 | 2 | 0 | 0 | 0 | 0 |
| G453C | 0 | 0 | 0 | 0 | 2 | 0 | 0 | 0 | 0 | 0 | 1 | 0 | 0 | 0 | 0 | 0 | 1 | 0 | 0 | 2 | 0 | 0 | 0 | 0 |
| **C469Y** | 0 | 2 | 4 | 0 | 4 | 0 | 0 | 0 | 0 | 0 | 0 | 1 | 0 | 3 | 0 | 0 | 2 | 3 | 0 | 9 | 0 | 0 | 1 | 0 |
| C471R | 0 | 0 | 0 | 0 | 0 | 1 | 0 | 0 | 2 | 0 | 0 | 1 | 0 | 0 | 3 | 0 | 0 | 1 | 0 | 0 | 0 | 0 | 0 | 0 |
| A504V | 0 | 0 | 0 | 0 | 2 | 0 | 0 | 0 | 0 | 0 | 0 | 0 | 0 | 0 | 0 | 0 | 0 | 0 | 0 | 3 | 0 | 0 | 1 | 0 |
| C489N | 0 | 2 | 0 | 0 | 5 | 1 | 0 | 0 | 2 | 0 | 0 | 0 | 0 | 0 | 1 | 0 | 0 | 19 | 0 | 3 | 1 | 0 | 2 | 0 |
| N499S | 0 | 0 | 0 | 0 | 1 | 0 | 0 | 1 | 0 | 0 | 0 | 0 | 0 | 0 | 0 | 0 | 1 | 0 | 0 | 2 | 0 | 0 | 0 | 0 |
| D501Y | 0 | 0 | 0 | 0 | 0 | 0 | 0 | 0 | 2 | 0 | 0 | 2 | 0 | 0 | 1 | 0 | 0 | 0 | 0 | 0 | 1 | 0 | 0 | 0 |
| V517I | 0 | 0 | 0 | 0 | 2 | 0 | 0 | 2 | 0 | 0 | 0 | 0 | 0 | 1 | 0 | 0 | 1 | 0 | 0 | 2 | 0 | 0 | 1 | 0 |
| S522C | 0 | 2 | 2 | 0 | 6 | 0 | 0 | 0 | 2 | 0 | 1 | 3 | 0 | 1 | 2 | 0 | 3 | 2 | 0 | 5 | 0 | 0 | 0 | 2 |
| N537S | 0 | 0 | 0 | 0 | 2 | 0 | 0 | 0 | 0 | 0 | 0 | 0 | 0 | 0 | 0 | 0 | 0 | 0 | 0 | 0 | 0 | 0 | 0 | 0 |
| **R561H** | 0 | 0 | 0 | 0 | 0 | 0 | 0 | 0 | 0 | 0 | 0 | 0 | 0 | 0 | 4 | 0 | 0 | 0 | 0 | 0 | 1 | 0 | 0 | 0 |
| R561C | 0 | 0 | 1 | 0 | 0 | 1 | 0 | 0 | 0 | 0 | 0 | 0 | 0 | 0 | 1 | 0 | 0 | 0 | 0 | 0 | 1 | 0 | 0 | 0 |
| A569S | 0 | 0 | 0 | 0 | 0 | 0 | 0 | 0 | 0 | 0 | 0 | 0 | 0 | 0 | 3 | 0 | 0 | 0 | 2 | 0 | 2 | 0 | 0 | 0 |
| A569T | 0 | 1 | 0 | 0 | 3 | 2 | 0 | 0 | 0 | 0 | 1 | 1 | 0 | 0 | 0 | 0 | 1 | 1 | 0 | 2 | 2 | 0 | 3 | 1 |
| A578S | 3 | 4 | 3 | 5 | 20 | 8 | 0 | 3 | 3 | 0 | 2 | 12 | 1 | 5 | 6 | 0 | 15 | 13 | 6 | 26 | 13 | 0 | 5 | 3 |
| P553S | 0 | 0 | 0 | 0 | 2 | 0 | 0 | 1 | 0 | 0 | 1 | 0 | 0 | 1 | 0 | 0 | 1 | 0 | 0 | 2 | 0 | 0 | 0 | 0 |
| **P553L** | 0 | 0 | 0 | 0 | 2 | 0 | 0 | 1 | 0 | 0 | 1 | 0 | 0 | 1 | 0 | 0 | 1 | 0 | 0 | 2 | 0 | 0 | 0 | 0 |
| Y558C | 0 | 0 | 0 | 0 | 0 | 0 | 0 | 0 | 0 | 0 | 2 | 0 | 0 | 2 | 0 | 0 | 4 | 0 | 0 | 4 | 0 | 0 | 1 | 0 |
| P667A | 0 | 0 | 1 | 0 | 0 | 0 | 0 | 0 | 0 | 0 | 0 | 0 | 0 | 0 | 0 | 0 | 0 | 0 | 0 | 0 | 0 | 0 | 0 | 0 |
| P667S | 0 | 1 | 1 | 0 | 0 | 1 | 0 | 1 | 10 | 0 | 1 | 0 | 0 | 0 | 2 | 0 | 2 | 1 | 0 | 2 | 3 | 0 | 1 | 0 |
| S679T | 0 | 1 | 0 | 0 | 0 | 0 | 0 | 2 | 0 | 0 | 0 | 0 | 0 | 0 | 0 | 0 | 0 | 0 | 0 | 0 | 0 | 0 | 0 | 0 |
| E691D | 0 | 9 | 0 | 0 | 24 | 0 | 0 | 7 | 0 | 0 | 5 | 0 | 0 | 10 | 0 | 0 | 7 | 0 | 0 | 32 | 0 | 0 | 4 | 0 |
| **A675V** | 0 | 0 | 6 | 0 | 0 | 7 | 0 | 0 | 11 | 0 | 0 | 9 | 0 | 1 | 1 | 0 | 0 | 4 | 0 | 0 | 9 | 0 | 0 | 1 |
| **Total SNPs** | **3** | **22** | **18** | **5** | **76** | **21** | **0** | **18** | **32** | **0** | **15** | **29** | **1** | **26** | **24** | **0** | **41** | **44** | **8** | **98** | **33** | **0** | **19** | **7** |

**Supplementary Table 2: Primer codon positions for *Pfk13* fragments and known artemisinin resistance mutations**

| **Fragment** | **Primer name** | **Primer sequence** | **Primer starting position** | **Primer ending position** | **Resistance associated mutations** |
| --- | --- | --- | --- | --- | --- |
| 1 | k13_extF_469 | CTTAACTTCTTAAGAAATCCG | codon 406 | codon 413 | 469Y, 553L, 561H |
|  | k13_extR_469 | AGGCATATGGAAATTGTTCCC | codon 612 | codon 617 |  |
|  | k13_intF_469 | GAAGCCTTGTTGAAAGAAGCAG | codon 426 | codon 432 |  |
|  | k13_intR_469 | CACCATTAGTTCCACCAATGAC | codon 589 | codon 595 |  |
| 2 | k13_extF_675 | GAAGCCTTG TTGAAAGAAGC | codon 426 | codon 431 | 553L, 561H, 675V |
|  | k13_extR_675 | CGGAGTGACCAAATCTGG | codon 716 | codon 720 |  |
|  | k13_intF_675 | GGGGGATATGATGGCTCTTCT | codon 544 | codon 550 |  |
|  | k13_intR_675 | ACTAATAAAGATGGGCCAAGC | codon 709 | codon 713 |  |
