# Supplementary material for: Serial cross-sectional school surveys identifies C469Y, P553L, R561H and A675V kelch 13 mutations associated with artemisinin resistance in Western Kenya
Source: Sci Rep. 2025 Nov 3;15:38303. doi: 10.1038/s41598-025-22286-7 (PMC12583745; doi:10.1038/s41598-025-22286-7)
Supplement: Supplementary file 2 — Supplementary Material 2 [file 41598_2025_22286_MOESM2_ESM.docx]

**Supplementary Figure 1:** Genomic mapping of *Pfk13* amplicon fragments to the coding region.

**
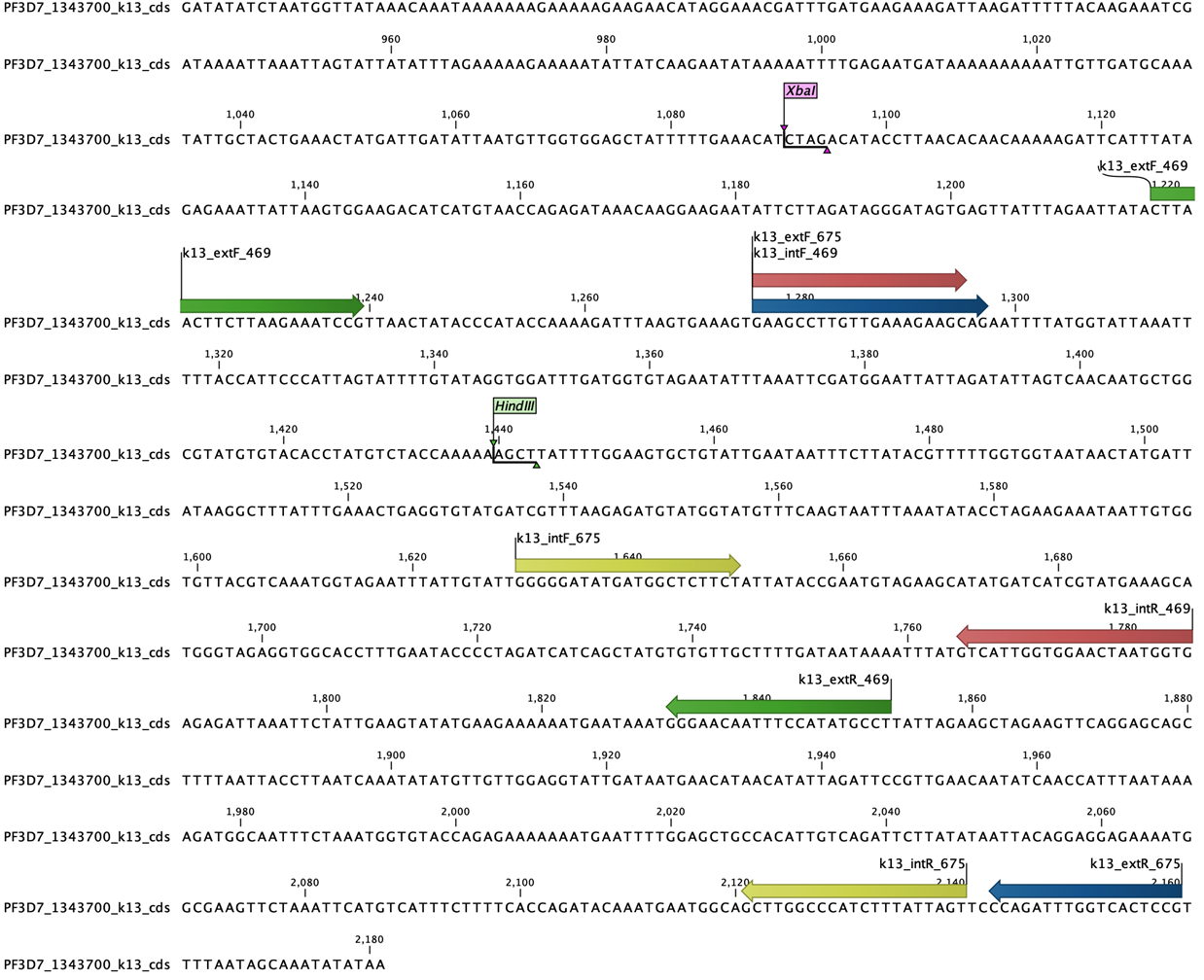
**

Arrows indicate the primers used, mapped to the reference sequence. Green denotes primers for the first fragment (k13_extF/R_469), red denotes internal primers for this fragment (k13_intF/R_469), blue denotes external primers for the second fragment (k13_extF/R_675), and yellow denotes internal primers for the second fragment (k13_intF/R_675).
